# GDF15 Promotes the Osteogenic Cell Fate of Periodontal Ligament Fibroblasts, thus Affecting Their Mechanobiological Response

**DOI:** 10.3390/ijms241210011

**Published:** 2023-06-11

**Authors:** Lukas Lösch, Albert Stemmler, Adrian Fischer, Julia Steinmetz, Lisa Schuldt, Christoph-Ludwig Hennig, Judit Symmank, Collin Jacobs

**Affiliations:** Department of Orthodontics, University Hospital Jena, Leutragraben 3, 07743 Jena, Germanychristoph-ludwig.hennig@med.uni-jena.de (C.-L.H.); collin.jacobs@med.uni-jena.de (C.J.)

**Keywords:** GDF15, orthodontic tooth movement, periodontal ligament fibroblasts, osteoblast differentiation, mechanobiological response, inflammation, osteoclast activity

## Abstract

Periodontal ligament fibroblasts (PdLFs) exert important functions in oral tissue and bone remodeling following mechanical forces, which are specifically applied during orthodontic tooth movement (OTM). Located between the teeth and the alveolar bone, mechanical stress activates the mechanomodulatory functions of PdLFs including regulating local inflammation and activating further bone-remodeling cells. Previous studies suggested growth differentiation factor 15 (GDF15) as an important pro-inflammatory regulator during the PdLF mechanoresponse. GDF15 exerts its effects through both intracrine signaling and receptor binding, possibly even in an autocrine manner. The extent to which PdLFs are susceptible to extracellular GDF15 has not yet been investigated. Thus, our study aims to examine the influence of GDF15 exposure on the cellular properties of PdLFs and their mechanoresponse, which seems particularly relevant regarding disease- and aging-associated elevated GDF15 serum levels. Therefore, in addition to investigating potential GDF15 receptors, we analyzed its impact on the proliferation, survival, senescence, and differentiation of human PdLFs, demonstrating a pro-osteogenic effect upon long-term stimulation. Furthermore, we observed altered force-related inflammation and impaired osteoclast differentiation. Overall, our data suggest a major impact of extracellular GDF15 on PdLF differentiation and their mechanoresponse.

## 1. Introduction

The orthodontic treatment of tooth malocclusions aims to improve and prevent medically relevant diseases of the teeth and supporting tissues such as the periodontium [1]. In addition to being highly prevalent in relation to the development of caries and periodontal inflammatory diseases, malocclusions and niches resulting from the crowding of teeth may also lead to root recession and tooth loss in the long term [2,3]. As a prophylactic therapy, orthodontic tooth movement (OTM) plays an important role in limiting the development and progression of these diseases. However, orthodontic therapy is associated with risks, including tooth root resorption, attachment, or even tooth loss [4,5,6]. Underlying causes increasing the occurrence of these risks appear to be multifactorial. In addition to genetic and epigenetic variations, changes in oral and systemic health, as well as treatment-specific conditions, are reported [7,8]. In particular, extrinsic factors including hormones and growth factors appear to influence the mechanofunctional remodeling processes of tissue and bone required for tooth movement [9].

The periodontal ligament (PdL) is a fibrous connective tissue located around the tooth root that serves to anchor the tooth in the alveolar bone. The PdL modulates the remodeling processes of tissue and bone induced by orthodontic forces leading to tooth movement [10]. The heterogeneous tissue includes diverse cell populations such as fibroblasts, osteoblasts, epithelial cell remnants of Malassez, macrophages, and undifferentiated mesenchymal stem cells [11]. The predominant cell type is PdL fibroblasts (PdLFs), which are characterized by many osteoblast-like properties such as the expression of osteogenic markers, e.g., alkaline phosphatase and osteocalcin, and are able to form mineral-like nodules in vitro [12,13,14].

Mechanical stimuli generated during OTM include tensile and compressive strains, which trigger the force-specific mechanobiological response of PdLFs to foster a favorable microenvironment for tissue and bone remodeling [15,16,17]. This includes the modulation of aseptic transient inflammatory responses by specific pro- and anti-inflammatory cytokines and the activation of bone-remodeling cells. In this context, the RANKL/RANK/OPG signaling pathway is particularly crucial as it modulates osteoclast differentiation and activity [18,19,20,21]. In response to specific stimuli, receptor activator of NF-kB ligand (RANKL) can be secreted by cells of osteogenic origin, stimulating the differentiation of osteoclast precursors by binding to their transmembrane-receptor RANK. Osteoclastogenesis can be blocked by binding RANKL to the decoy receptor osteoprotegerin (OPG), which is secreted by osteoblast-like cells. Unfavorable RANKL/OPG values are not only relevant in terms of bone remodeling defects but also in root resorption as well as tooth attachment or total tooth loss [22].

Tensile forces promote bone formation by increasing the secretion of the anti-inflammatory interleukin IL-10 and stimulating osteoblast differentiation [23,24]. Due to the intrinsic potential of PdL fibroblasts to differentiate into osteoblasts, the increased expression of osteogenic markers such as alkaline phosphatase (ALP) and runt-related transcription factor 2 (RUNX2), as well as increased calcium deposits, were detected upon tensile force application [25,26]. Contrarily, compression promotes a bone-resorbing microenvironment by inducing hypoxia and the secretion of pro-inflammatory cytokines including IL-6, IL-8, and prostaglandin E2 (PGE2), as well as RANKL, by PdLFs [27,28,29,30].

As a stress-responsive multifunctional regulator [31,32], we have recently identified growth differentiation factor 15 (GDF15) as an important pro-inflammatory modulator in the mechanoresponse of PdLFs to compressive forces [33,34]. As a member of the transforming growth factor-β (TGF-β)/bone morphogenic protein (BMP) superfamily [31], GDF15 exerts important functions in the regulation of various cellular processes [32,35]. Synthesized as a precursor protein, GDF15 undergoes disulfide-linked dimerization prior to secretion [36]. Extracellular GDF15 can bind to cell membrane receptors, with the α-like receptor of the GDNF family (GFRAL) being the best-characterized receptor exclusively expressed in the central nervous system [37]. Additionally, it has been reported that members of the activin receptor-like kinase (ALK) family also bind GDF15 and mediate autocrine and paracrine functions [38,39,40]. In addition to its receptor-mediated signaling, unprocessed GDF15 has also been reported to modulate gene expression after translocation into the nucleus [41,42].

GDF15 serum levels are highly elevated in association with various diseases including obesity and cancer as well as aging and have been implicated as potential biomarkers [43,44]. Considering its crucial role in the mechanoresponse of PdL fibroblasts [34,45], prolonged exposure to GDF15 could impair their functions and thus increase the risks for these patients in the context of orthodontic therapy. This seems particularly relevant considering that GDF15 has been shown to modulate osteoblast differentiation and function [46,47], potentially altering cell fate and the mechanoresponse of PdLFs. Therefore, this study aims to investigate the effect of prolonged GDF15 exposure on the cellular properties and mechanobiological functions of PdL fibroblasts.

## 2. Results

### 2.1. hPdLFs Express ALK1, ALK2, and ALK5

**Figure 1:**
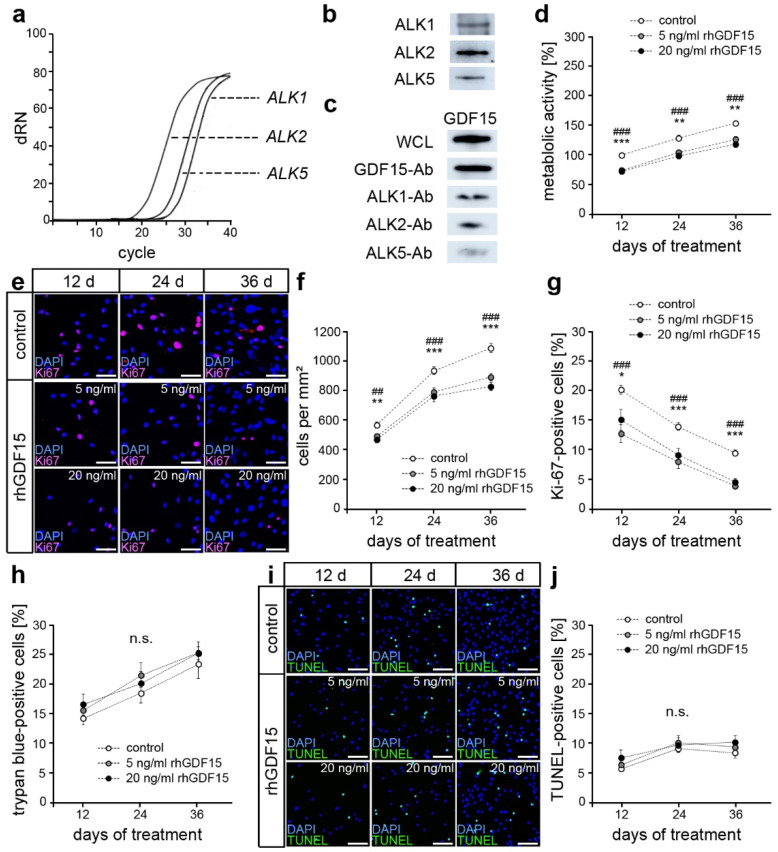
GDF15 limits cell proliferation of hPdLFs without affecting cell survival in the long term. (**a**,**b**) RNA (**a**) and protein (**b**) expression of the activin receptor-like kinases (ALK1, 2, and 5) in hPdLFs. (**c**) Co-immunoprecipitation with ALK1, ALK2, and ALK5-specific antibodies (ALK1/2/5-Ab) to detect GDF15 binding by those receptors in hPdLFs stimulated with 5 ng/mL recombinant human GDF15 (rhGDF15). GDF15 detection in whole cell lysates (WCLs) and GDF15-Ab-precipitated proteins were used as positive controls. (**d**) Metabolic activity of hPdLFs after stimulation with 5 ng/mL and 20 ng/mL recombinant human GDF15 (rhGDF15) for 12, 24, and 36 days displayed in relation to the 12-day control. (**e**–**g**) Ki-67-positive hPdLFs (magenta) after stimulation with rhGDF15 with cell nuclei (blue; (**e**)). The number of cells per mm^2^ is displayed in (**f**), and (**g**) shows the proportion of Ki-67-positive hPdLFs. (**h**) The number of trypan blue-positive hPdLFs after rhGDF15 stimulation, indicating the proportion of dead cells. (**i**,**j**) TUNEL-positive hPdLFs (green, (**i**)) after rhGDF15 stimulation with cell nuclei (blue), displayed as a proportion to the cell number (**j**). * *p* < 0.05; **/## *p* < 0.01; ***/### *p* < 0.001; */**/*** control in relation to 5 ng/mL rhGDF15; ##/### control in relation to 20 ng/mL rhGDF15; one-way ANOVA and Tukey post hoc test. Scale bar: 20 µm in (**e**) and 25 µm in (**i**); d, days; dRN, the difference between baseline and measured fluorescence; n.s., not significant.

Considering the neuron-specific expression of the best-characterized GDF15 receptor GFRAL, ALK1, ALK2, and ALK5 were recently reported as potential receptors in other cell types [38,39,40]. Using quantitative PCR, we detected gene expression of those ALKs also in hPdLFs (Figure 1a). A Western blot analysis confirmed their expression at the protein level (Figure 1b). Next, we performed co-immunoprecipitation with specific antibodies against ALK1, ALK2, and ALK5 (ALK1/2/5-Ab) to detect receptor binding of GDF15 in hPdLFs. To this end, we stimulated hPdLFs for 24 h with recombinant human GDF15 (rhGDF15, 5 ng/mL; Figure 1c), which represents a biologically active, disulfide-linked homodimer of matured GDF15. This indicated the binding of GDF15 to all the three ALKs examined, suggesting them as potential GDF15 receptors in PdL fibroblasts as well. Of note, the GDF15 detection antibody was also used for precipitation (GDF15-Ab) to serve as a positive control. Furthermore, GDF15 was detected in whole cell lysates (WCLs) of rhGDF15-stimulated hPdLFs.

### 2.2. GDF15 Exposure Limits Cell Proliferation without Affecting the Survival of hPdLFs

An extended GDF15 exposure might occur due to high serum levels in different diseases including obesity and cancer as well as in aging. To evaluate the impact of prolonged GDF15 exposure on hPdLFs, we first analyzed metabolic activity after 12, 24, and 36 days of stimulation with rhGDF15 (Figure 1d). We utilized two concentrations of rhGDF15, 5 ng/mL and 20 ng/mL, which are in the normal to high range of circulating GDF15 detected in the serum levels of several diseases [43,48]. A 12-day stimulation led to reduced metabolic activity, which was sustained with prolonged stimulation.

Since changes in metabolic activity may be based on differences in cell number and thus proliferation level, we subsequently determined cell density (Figure 1e,f). After 12 days of stimulation, a significant decrease in cell density was observed, which became even more pronounced with increasing duration of exposure, but was independent of the applied concentration of rhGDF15. To determine the proportion of proliferative cells, immunofluorescence staining of the proliferation marker Ki-67 was performed after 12, 24, and 36 days of rhGDF15 exposure (Figure 1e,g). Under all conditions, a decreasing proportion of Ki67-positive cells was observed over the culture period, resulting from either growth senescence due to limited space or the induction of differentiation programs. However, this decrease was significantly stronger in rhGDF15-stimulated hPdLFs, resulting in the lowest number of Ki-67-positive cells after 36 days of exposure compared to the control. A significant difference between both concentrations of the applied rhGDF15 could not be detected at any of the analyzed time points.

Since GDF15 was reported to affect cell survival, we additionally analyzed the proportion of damaged cells using trypan blue staining (Figure 1h) and apoptosis using the TUNEL assay (Figure 1i,j), respectively. However, we neither detected changes in cell survival in relation to culture duration nor caused by exposure to rhGDF15. Together, GDF15 limits the proliferative capacity of hPdLFs without affecting cell survival.

### 2.3. Long-Term Exposure to GDF15 Promotes Osteogenic Cell Fate of hPdLFs

**Figure 2:**
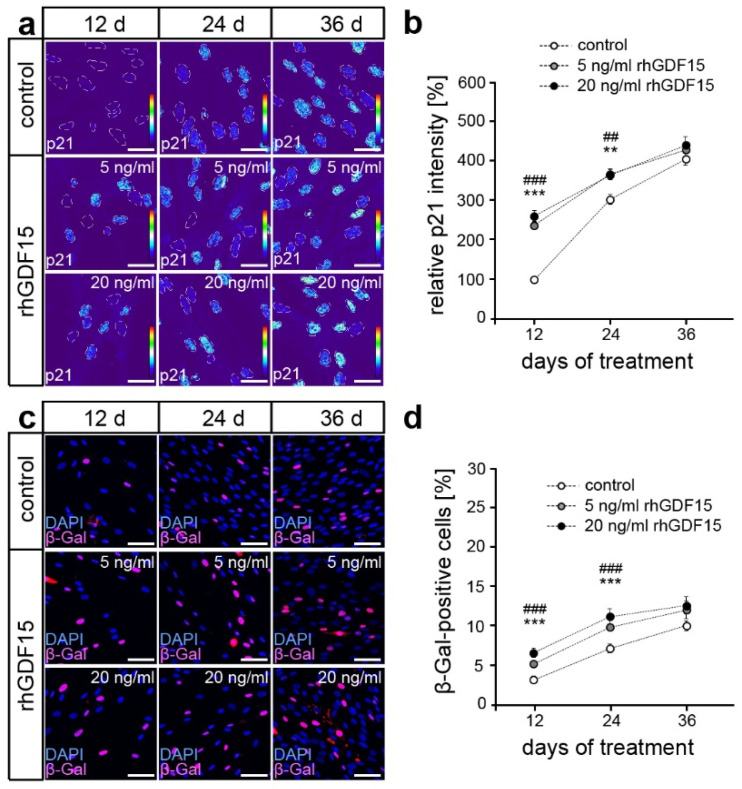
GDF15 promote cellular senescence of hPdLFs. (**a**,**b**) p21 intensity in hPdLFs stimulated with 5 ng/mL and 20 ng/mL rhGDF15 for 12, 24, and 36 days shown as thermal LUT (**a**). Grey scattered lines surround the cell nuclei. Mean p21 intensity is shown in relation to the 12-day control in (**b**). (**c**,**d**) β-galactosidase (β-Gal)-positive hPdLFs (magenta) after rhGDF15 stimulation with cell nuclei (blue, (**c**)). The proportion of β-Gal-positive hPdLFs is displayed in (**d**). **/## *p* < 0.01; ***/### *p* < 0.001; **/*** control in relation to 5 ng/mL rhGDF15; ##/### control in relation to 20 ng/mL rhGDF15; one-way ANOVA and Tukey post hoc test. Scale bar: 10 µm in (**a**) and 25 µm in (**c**); d, days.

Since cell survival was not affected, the reduced proliferation of hPdLFs might be caused by increased cellular senescence or osteogenic differentiation, which were both shown to be potentially affected by GDF15 [49,50]. Quantitative analysis of the immunofluorescent staining intensity of p21, an early senescence marker, revealed significantly higher expression levels after 12 and 24 days of rhGDF15 stimulation at both concentrations (Figure 2a,b). However, this increase was undetectable at the latest point of stimulation, where p21 levels were not significantly different from the corresponding control. These findings were confirmed using an analysis of β-galactosidase activity (Figure 2c,d). Thus, our data suggest enhanced activation of cellular senescence after 12 days of stimulation with rhGDF15, whereas no significant changes were observed with prolonged exposure.

**Figure 3:**
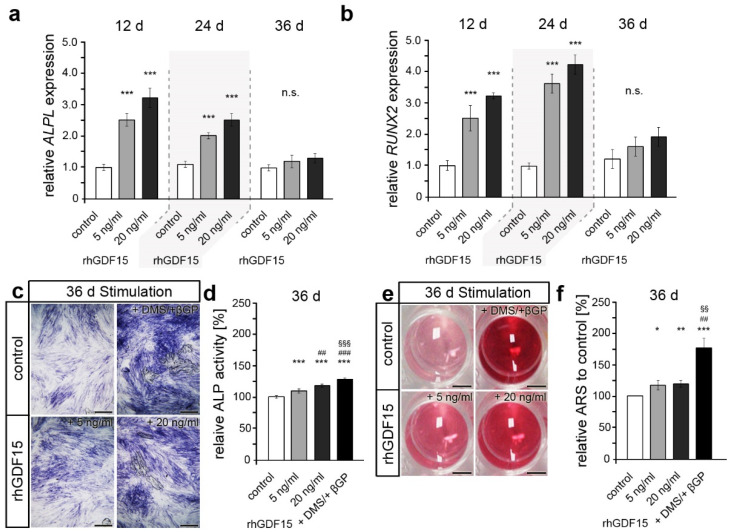
Long-term exposure to GDF15 fosters the osteogenic differentiation of hPdLFs. (**a**,**b**) *ALPL* and *RUNX2* expression encoding osteoblast-related markers in hPdLFs stimulated with 5ng/mL and 20 ng/mL recombinant human GDF15 (rhGDF15) for 12, 24, and 36 days displayed in relation to the control at each time point. (**c**,**d**) ALP activity (dark blue; (**c**)) of 36-day rhGDF15-stimulated hPdLFs displayed in relation to the control in (**d**). (**e**,**f**) Alizarin red staining intensity of hPdLFs in wells stimulated for 36 d with rhGDF15 (**e**) displayed as a percentage (%) in relation to the control in (**f**). Stimulation with DMS and βGP was used as a positive control for osteogenic differentiation. * *p* < 0.05; **/##/§§ *p* < 0.01; ***/###/§§§ *p* < 0.001; */**/*** in relation to control; ##/### in relation to 5 ng/mL rhGDF15; §§/§§§ in relation to 20 ng/mL rhGDF15; one-way ANOVA and Tukey post hoc test. Scale bar: 25 µm in (**c**), 5 mm in (**e**); ARS, alizarin red staining; d, days; n.s., non significant.

In the following, we aimed to address the osteogenic differentiation and potential alterations by GDF15, which may be a significant contributing factor to the diminished proliferative capacity of stimulated hPdLFs apart from senescence. To this end, quantitative expression analysis of the osteogenic marker genes *ALPL* and *RUNX2* were performed and showed increased levels after 12 and 24 days of rhGDF15 exposure (Figure 3a,b). However, compared to the corresponding control, no difference in osteogenic gene expression was detected at the latest time point. Further analysis of alkaline phosphatase activity as a characteristic of osteogenic differentiation clearly showed enhanced levels after 36 days of rhGDF15 exposure (Figure 3c,d). We used hPdLFs stimulated with dexamethasone and β-glycerol phosphate for 36 days as an osteogenic differentiation control [51]. Of note, RNA transcription levels may not necessarily correlate with respective protein levels and post-translational regulation may additionally modulate protein activity [52].

Considering that ALP activity is critical for mineralization, we further analyzed calcium deposit formation as a marker for active osteoblasts at 36 days of rhGDF15 stimulation (Figure 3e,f). Likewise, as noted for ALP activity, we observed a slightly increased formation of mineralized deposits upon long-term stimulation with rhGDF15, which also appeared to be concentration-independent. However, the increase was significantly higher for the osteogenic differentiation control.

Taken together, our data suggest that GDF15 promotes osteogenic differentiation of hPdLFs upon long-term exposure, whereas cellular senescence is more prominent with shorter stimulation periods.

### 2.4. Prior Long-Term Exposure of hPdLFs to rhGDF15 Affects Their Mechanoreactivity

**Figure 4:**
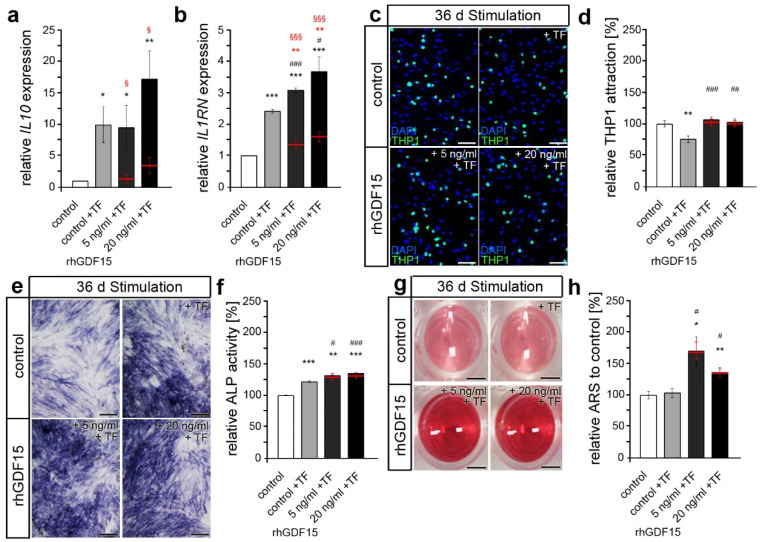
Long-term GDF15-exposed hPdLFs show a reduced anti-inflammatory and pro-osteogenic mechanoresponse to tensile forces. (**a**,**b**) Quantitative expression levels of *IL10* (**a**) and *IL1RN* (**b**) encoding anti-inflammatory markers in hPdLFs stimulated with 5ng/mL and 20 ng/mL recombinant human GDF15 (rhGDF15) for 36 days and stressed with tensile forces for 24 h (+TF) displayed in relation to the untreated control. (**c**,**d**) Adherent (activated) THP1 cells (green) on stimulated and stressed hPdLFs (blue, cell nuclei) displayed as the number of THP1 cells per hPdLFs and in relation to the untreated control in (**d**). (**e**,**f**) ALP activity (dark blue) of 36-day rhGDF15-stimulated hPdLFs stressed with TF (c), displayed in relation to the control in (**d**). (**g**,**h**) Alizarin red staining intensity of hPdLFs in wells stimulated for 36 d with rhGDF15 and stressed with TF (**g**), displayed as a percentage (%) in relation to the control in (**h**). Stimulation with DMS and βGP was used as a positive control for osteogenic differentiation. Red lines show baseline levels of the respective condition. */#/§ *p* < 0.05; **/## *p* < 0.01; ***/###/§§§ *p* < 0.001; */**/*** in relation to control; #/##/### in relation to control + TF; §§§ baseline (red) in relation to 20 ng/mL rhGDF15 + TF; one-way ANOVA and post hoc test (Tukey). Scale bars: 25 µm in (**c,e**), 5 mm in (**g**); ARS, alizarin red staining; d, days.

In the following, we aimed to determine the extent to which the shift towards an osteogenic cell fate by prolonged rhGDF15 exposure for 36 d affects the functionality of hPdLFs responding to mechanical stimuli. To this end, we first applied biaxial tensile force, typically inducing anti-inflammatory responses and activating osteoblast differentiation, to foster bone formation. Subsequent analysis of genes encoding the anti-inflammatory markers IL-10 (*IL10*; Figure 4a) and IL-1RA (*IL1RN*; Figure 4b), which are increased in hPdLFs following tensile forces [53], revealed no relevant changes due to prior prolonged rhGDF15 exposure. Even though the expression of *IL1RN* appears increased in GDF15-stimulated fibroblasts after tensile force, the baseline levels (red lines) are already enhanced, resulting in comparable fold changes in all conditions to the corresponding tensile-stressed cells (control: 2.42 ± 0.06; 5 ng/mL rhGDF15: 2.21 ± 0.07, *p*-value to control 0.99847; 20 ng/mL rhGDF15: 2.69 ± 0.09, *p*-value to control 0.98791).

We next analyzed adherent THP1 cells used to visualize the inflammatory response by determining the activation of monocytic immune cells (Figure 4c,d). Whereas tensile force promoted an anti-inflammatory response with reduced THP1 activation, prior rhGDF15 exposure blocked this response.

Since bone formation and the activation of osteoblast activity are key features of the tensile site, we further analyzed ALP activity induction in hPdLFs after 24 h of biaxial tensile stress (Figure 4e,f). Whereas the control treatment displayed increased ALP activity due to the application of tensile force, this was not detected for rhGDF15-stimulated hPdLFs, pointing to a limited activation of further osteoblasts by tensile stress. Of note, ALP activity in rhGDF15-exposed stretched hPdLFs was still significantly higher than in stressed control cells due to increased baseline levels (Figure 4f; 5 ng/mL rhGDF15 to control: *p*-value 0.0001 × 10^27^, ***; 20 ng/mL rhGDF15 to control: *p*-value 0.0002 × 10^5^, ***).

Furthermore, we examined the formation of calcium deposits after the application of tensile stress (Figure 4g,h). While the previously detected GDF15-dependent increase in calcium deposit formation was also detected in force-stressed cells, neither the control cultures nor rhGDF15-stimulated hPdLFs showed tensile-related differences compared to unstressed hPdLFs. Due to the relatively slow process of calcium deposition, this might be expectable in regard to the short duration of additional mechanical stress application.

Based on its pivotal role in modulating the pro-inflammatory response to compressive stimuli [34], we next examined the effects of prior long-term stimulation on the mechanoresponse of compressed hPdLFs. Compressive forces promote pro-inflammatory responses in hPdLFs and facilitate bone degradation by increasing RANKL levels and activating osteoclasts.

**Figure 5:**
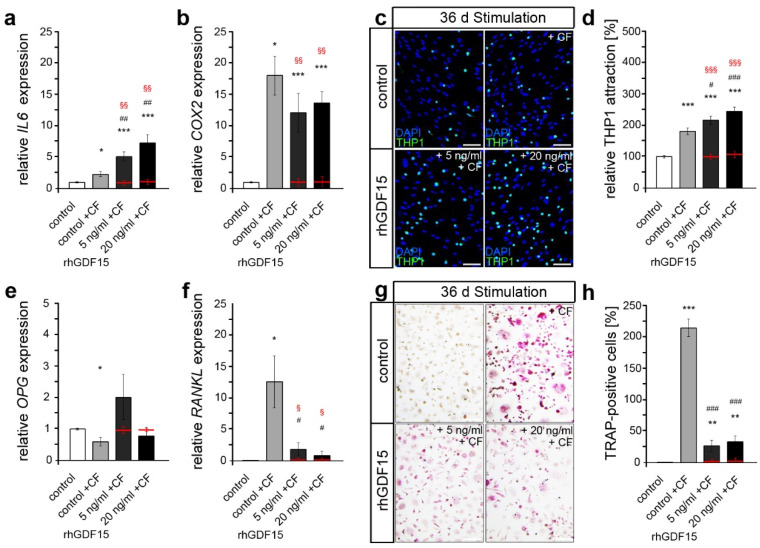
Long-term GDF15-exposed hPdLFs show an increased pro-inflammatory response to compressive stimuli with limited activation of osteoclasts. (**a**,**b**) Quantitative expression levels of *IL6* (**a**) and *COX2* (**b**) encoding pro-inflammatory markers in hPdLFs stimulated with 5 ng/mL and 20 ng/mL recombinant human GDF15 (rhGDF15) for 36 days and stressed with compressive forces for 24 h (+CF), displayed in relation to the untreated control. (**c**,**d**) Adherent (activated) THP1 cells (green) on stimulated and stressed hPdLFs (blue, cell nuclei) displayed as the number of THP1 cells per hPdLFs and in relation to the untreated control in (**d**). (**e**,**f**) Quantitative expression levels of *OPG* (**e**) and *RANKL* (**f**) in stimulated and stressed hPdLFs displayed in relation to the untreated control. (**g**,**h**) TRAP-positive THP1 cells (magenta; (**g**)) indicating the differentiation into osteoclasts, when stimulated with the medium supernatant of 36-day rhGDF15-stimulated hPdLFs additionally stressed with CF. The proportion of TRAP-positive osteoclasts is displayed in (**h**). Red lines show baseline levels of the respective conditions. */#/§ *p* < 0.05; **/##/§§ *p* < 0.01; ***/###/§§§ *p* < 0.001; */**/*** in relation to control; #/##/### in relation to control + TF; §§/§§§ baseline (red) in relation to 5 ng/mL or 20 ng/mL rhGDF15 + TF; one-way ANOVA and Tukey post hoc test. Scale bars: 25 µm in (**c**,**g**); d, days.

First, we performed quantitative expression analysis on important genes encoding the pro-inflammatory cytokines IL-6 (*IL6*) and COX2 (Figure 5a,b). Whereas no GDF15-dependent changes were detected in the expression of *COX2*, *IL6* levels were significantly increased in compressed rhGDF15-stimulated hPdLFs. Of note, baseline levels were not changed with rhGDF15 exposure.

To investigate whether those changes in *IL6* expression also cause an altered inflammatory response and thus activation of immune cells, we analyzed the adhesion of THP1 monocytic cells accordingly (Figure 5c,d). Thus, compared with controls, increased activation of monocytic cells by compressed hPdLFs was detected during long-term stimulation with rhGDF15.

We next focused on a possible impact on the activation of bone-resorbing osteoclasts by analyzing *RANKL* and *OPG* expression of force-stressed hPdLFs, which encode key players in the regulation of osteoclastogenesis (Figure 5e,f). The application of compressive force promoted a pronounced increase in *RANKL* levels and a significant decrease in *OPG* levels in controls, which is typically seen under this condition to promote osteoclast differentiation by increasing RANKL-RANK binding [54]. However, these changes were not observed in rhGDF15-stimulated hPdLFs.

To further functionally address changes in osteoclast activation, macrophages resulting from pre-stimulated THP1 monocytic cells were cultured for six days with the medium supernatant of compression-stressed hPdLFs, which includes secreted proteins such as RANKL to stimulate their differentiation into osteoclasts [55] (Figure 5f,g). Using a TRAP assay, enhanced differentiation into mononuclear pre-osteoclasts and matured multinuclear osteoclasts was detected after stimulation with the medium supernatant of compressed control fibroblasts compared to the control that was not stressed with compressive force. Of note, this unstressed control also served as a negative control, since RANKL levels were expected to be low here. However, consistent with the RANKL and OPG expression data, decreased osteoclast activation was observed in compressed hPdLFs extensively exposed to rhGDF15. However, it was still enhanced compared to the control and the rhGDF15-stimulated fibroblasts (red lines) that were not mechanically compressed.

Together, our data indicate a relevant impact of long-term stimulation with GDF15 on the cellular properties and the pro-inflammatory mechanobiological response of human PdL fibroblasts, resulting in increased inflammation due to compressive forces but reduced osteoclast activation, potentially via modulation of RANKL/OPG.

## 3. Discussion

The interdependent and interrelated responses of periodontal ligament cells to mechanical forces generated during orthodontic treatment are modulated by a variety of key factors [27,56]. A change in their balance or functionality due to extrinsic and intrinsic factors can affect tooth movement. Recently, we demonstrated a relevant pro-inflammatory role of GDF15 in the mechanosignaling of PdLFs [34]. Elevated GDF15 serum levels have been detected in various diseases and within the orthodontic context, more relevant in obesity and aging [44,57], which may impact tooth movement in these patient groups.

Thus, to uncover the potential effects of an extended GDF15 exposure on the mechanofunctionality of periodontal cells, we stimulated PdL fibroblasts for up to 36 days with recombinant GDF15 protein prior to subsequent analysis of the response to mechanical forces. GDF15 stimulation resulted in significantly decreased proliferation correlating with an increased rate of cellular senescence at shorter exposures, while longer stimulation resulted in a more prominent level of osteogenic differentiation. Surprisingly, compressed hyperinflammatory PdLFs did not display correlated excessive osteoclast activation; in fact, it was significantly attenuated due to long-term GDF15 exposure.

GDF15 is known for its multiple functions in tissue proliferation and differentiation and has also been detected in fibroblasts. Kim et al. demonstrated an inhibiting effect of GDF15 on renal fibroblast growth in primary fibroblasts isolated from mouse kidneys subjected to ureteral obstruction-induced fibrosis [58]. In addition, decreased growth and activation of lung fibroblasts due to GDF15-associated alterations in the TGF-Smad pathway have been described [59]. Contrarily, Guo et al. recently revealed that GDF15 can promote proliferation in newborn rat cardiac fibroblasts after irradiation, which was confirmed by the increased cell proliferation rate and increased expression of fibrosis markers (Col1α and αSma) after transfection with GDF15 in vitro [60]. However, our data support an antiproliferative effect of GDF15, specifically on hPdLFs, in terms of decreased Ki67, whereas we could not detect an impact on the apoptosis rate of hPdLFs.

We presumed that increased cellular senescence and osteogenic differentiation could be reasons for the antiproliferative effect of GDF15 on hPdLFs. GDF15 is highly upregulated with age and associated with many age-related diseases [61]. It is thus suggested as a biomarker for aging [44]. Moreover, GDF15 levels correlate positively with cellular senescence induced using ionizing radiation in human aortic endothelial cells [49]. However, the pleiotropic regulator GDF15 is also associated with anti-senescence effects. Thus, Li et al. reported that GDF15 downregulates p21, a senescence marker, via activation of both the PI3K/AKT and MAPK/ERK signaling pathways, resulting in cervical cancer cell proliferation [62]. We detected higher p21 levels and increased ß-galactosidase activity due to GDF15 stimulation in hPdLFs, supporting the function of GDF15 as a promoter of cellular senescence.

Apart from cellular senescence, we considered osteogenic differentiation as a potential reason for the GDF15-related reduction in hPdLF proliferation. In this study, we provide supporting evidence regarding the osteogenic potential of GDF15 in PdL fibroblasts. Quantitative expression analysis of osteogenic marker genes *ALPL* and *RUNX2* showed increased levels after 12 and 24 days of rhGDF15 exposure. ALP activity increased accordingly but showed a temporal shift compared to the expression pattern with its highest activity at 36 days of exposure. We further detected an increase in mineralized deposits of rhGDF15 exposed cells compared to the control condition, which undermines the osteogenic effect of GDF15.

Members of the TGF-ß family are known to regulate osteoblast and osteoclast differentiation [63,64]. In addition to our own previous results in primary osteoblasts [33], a pro-osteogenic effect of GDF15 on various cell types has been observed in a number of studies. Uchiyama et al. demonstrated that the number of bone marrow-derived mesenchymal stem cells (BM-MSCs) increased significantly after 7 days of stimulation with rhGDF15 [50]. Further analysis showed that the protein levels of representative markers for osteoblastic differentiation RUNX2 and OSX were increased in rhGDF15-treated human BM-MSCs compared to control cells. Moreover, transfection with *GDF15* cDNA was shown to promote osteoblast differentiation in prostate cancer cells in vitro [65]. According to Siddiqui et al. [47], prostate cancer (PCa)-secreted GDF15 promotes bone metastases and bone turnover. Further, rhGDF15 promotes osteogenic differentiation of mouse calvarial osteoblasts and GDF15 deletion inhibits PCa-mediated osteoblast differentiation and mineralization, suggesting an essential role of GDF15 in the stimulation of osteoblast differentiation by PCa. It should be noted that Westhrin et al. also reported an anti-osteogenic effect of GDF15 on human bone marrow-derived mesenchymal stem cells with reduced alkaline phosphatase activity, matrix mineralization, and mRNA levels of *RUNX2*, Type I collagen, and osteocalcin due to GDF15 stimulation for 17 days [46]. However, altered cell fate might influence cellular responses to mechanical forces.

We recently reported that *GDF15*-deficient hPdLF showed a significantly reduced secretion of the pro-inflammatory cytokines IL6, PGE2, and TNFα upon mechanical compression [34]. Combined with lower numbers of activated monocytic THP1 cells, our previous data suggest a pro-inflammatory role for GDF15 in hPdLFs. However, we left one relevant question unexplored. What is the role of intracellular GDF15 activity versus its effects by extracellular stimulation through receptor binding? At different protein states, GDF15 can trigger intracrine signaling by translocation to the nucleus and auto- or paracrine signaling through membrane-bound receptors [39,41]. Artz et al. identified ALK-5 as a receptor expressed in mouse leucocytes that inhibits integrin activation triggered by GDF15 [40]. Moreover, GDF15 has been found to trigger analgesia in rat primary sensory neurons via the ALK-2 receptor [39] and functions as a potential GDF15 receptor in human airway epithelial cells [38]. We have now demonstrated for the first time that hPdLFs also express ALK1, ALK2, and ALK5, as well as that they at least bind extracellular biologically active GDF15 homodimers in this cell type.

Extending our previous study, our new data suggest that the pro-inflammatory effect of GDF15 in compressed hPdLFs is mediated by auto- or paracrine action via protein-receptor binding. Thus, increased IL6 levels and activated monocytes were detected due to GDF15 stimulation. Interestingly, GDF15-stimulated mechano-controls showed comparable transcription levels of *IL6*, which is in contradiction to Li et al., who reported increased *IL6* expression levels in unforced hPdLFs due to GDF15 exposure [45]. A plausible explanation might relate to the concentration of GDF15 used for cell exposure. While we stimulated with 5 to a maximum of 20 ng/mL GDF15 protein, which corresponds to high concentrations secreted by various cells upon different stresses [66,67,68,69,70], Li et al. used an even higher concentration of 100 ng/mL. Nevertheless, both studies confirm a pro-inflammatory influence of GDF15 in hPdLFs.

Compressed fibroblasts usually show an increased RANKL and a decreased OPG secretion leading to enhanced osteoclast activation [71,72,73]. Our data indicate that long-term exposure to 5–20 ng/mL rhGDF15 significantly reduces those mechano-related effects as reduced *RANKL/OPG* expression ratios and osteoclast activation were detected. In contrast to this, short-term stimulation with a significantly higher dose of 100 ng/mL GDF15 protein resulted in an increased *RANKL/OPG* expression ratio in human PdL cells mainly due to reduced OPG levels [45]. Since we stimulated cells with GDF15 for 36 days driving an osteogenic cell fate, the mechanoreactivity might differ due to cell differentiation. Comparable to our results, GDF15 stimulation of mouse calvarial osteoblasts (MCOs) also failed to induce changes in *Opg* expression levels [47]. In contrast to hPdLFs, GDF15-exposed MCOs showed increased *Rankl* levels when stimulated with GDF15 concentrations higher than 10 ng/mL. However, osteoclast activation was not functionally validated in both previous studies within this context [45,47], which limits comparison with our study in terms of possible differences between RNA and protein expression as well as secretion. Furthermore, considering the limited sequence homology of GDF15 between humans and rodents [74], possible effects of non-species-matched stimulation may account for differences in the findings of Siddiqui, who used human recombinant GDF15 to stimulate mouse MCOs. Nevertheless, the GDF15 stimulation of PdL fibroblasts and (PdL-derived) osteoblasts appears to affect the activation of osteoclasts by these cells, although this seems to depend on the specific conditions.

In general, stimulation studies with GDF15 are limited by its relatively short half-life in circulating systems due to rapid renal clearance as well as due to protein stability [48]. This might affect study outcomes as well as the interpretation and comparability of results. However, Kempf et al. also demonstrated that GDF15 concentrations in blood samples remained relatively stable over 48 h at room temperature in terms of immunoreactivity [75]. One could, of course, speculate on the extent to which its biologically active form is retained. We stimulated with 5 ng/mL and 20 ng/mL of biologically active rhGDF15 every 48 h. Since we used concentrations in the upper range of circulating GDF15 levels commonly measured in vivo in various diseases [43,48], we assume that this provided sufficient and reliable stimulation, even if it probably diminished over the 48 h. However, for future studies, particularly regarding the transferability to in vivo models, we would recommend long-acting GDF15 molecules and analogs [48,76].

In summary, our results strongly suggest a relevant impact of GDF15 on the cell fate of PdL fibroblasts promoting osteogenic differentiation over an extended period. Moreover, GDF15 seems to impact the force-related inflammatory mechanoresponse of those cells and subsequently affected their activation of osteoclasts. Since we used a simplified in vitro model to simulate orthodontic tooth movement with compressive and tensile strain, future studies should focus on these findings in vivo. Altogether, our study provides evidence that long-term elevated GDF15 levels, such as those associated with specific diseases as well as with aging [43,44], indeed impact the mechanoreactivity of PdL cells and thus may be relevant in the orthodontic treatment of those patients.

## 4. Materials and Methods

### 4.1. Cell Culture

Human periodontal ligament fibroblasts (hPdLFs, Lonza, Basel, Switzerland) were cultured in culture medium containing Dulbecco’s modified Eagle’s medium (DMEM, Capricorn Scientific, Ebsdorfergrund, Germany), 4.5 g/L Glucose, 10% heat-inactivated fetal bovine serum (Thermo Fisher Scientific, Carlsbad, CA, USA), 100 U/mL penicillin, 100 mg/L streptomycin, and 50 mg/L L-ascorbic acid at 37 °C, 5% CO_2_, and 95% humidity. Cells were passaged at 75% confluence with 0.05% Trypsin/EDTA (Thermo Fisher Scientific, Carlsbad, CA, USA). Cells from passages six to nine were used for the experiments.

THP1 monocytic cells (DMSZ, Braunschweig, Germany) were cultured at 37 °C, 5% CO_2_, and 95% humidity in RPMI 1640 medium (Thermo Fisher Scientific, Carlsbad, CA, USA) containing 10% FBS, 100 U/mL penicillin, and 100 g/mL streptomycin. Cells were passaged weekly and seeded at a density of 1 × 10^6^ cells in a T175 culture flask (Thermo Fisher Scientific, Carlsbad, CA, USA).

### 4.2. Stimulation with Recombinant Human GDF15 Protein

For evaluation of GDF15-induced effects, hPdLFs were stimulated for 12, 24, and 36 days with 5 ng/mL or 20 ng/mL recombinant human GDF15 protein (rhGDF15, 9279-GD-050, R&D Systems, Minneapolis, MN, USA) in culture flasks. This is a carrier-free, disulfide-linked homodimer of matured GDF15, which is produced in Escherichia coli and is biologically active. According to the manufacturer’s instructions, the protein was reconstituted in 4 mM HCl. HCl-containing controls were used, respectively. As an osteoblast differentiation control, hPdLFs were stimulated with 100 nM dexamethasone and 10mM β-glycerol phosphate. Twelve days prior to the final experiments, 100,000 cells were seeded into 6-well plates. For immunofluorescent analysis and determination of THP1 activation, 5000 cells were seeded onto glass coverslips in 48-well plates and further cultured for 12 days in respective media.

### 4.3. Application of Mechanical Forces

On the final day of stimulation, medium including rhGDF15 was freshly applied and static compression of 2 g/cm^2^ was achieved using the application of sterilized glass plates for 24 h according to the protocol of Kirschneck et al. [77] and as described before [33,34,78]. For subsequent analysis of monocyte activation, force application was performed in 24-well plates using centrifugation for two rounds of twelve-hour centrifugations at 30 °C with a force of 7.13 g/cm^2^. Control cells were cultured at 30 °C for the duration of the force application. A three-hour recovery break at 37 °C, under 5% CO_2_, and 95% humidity was applied between the centrifugation rounds.

For the application of static isotropic tensile force, hPdLFs were seeded on flexible bottomed 6-well plates (BioFlex^®^ Culture Plates, FLEXCELL^®^, Asbach, Germany) coated with pronectin. Tensile forces of 15.9% were applied using spherical cap silicone stamps of a 25 mm radius and a height of 7.1 mm according to Nazet et al. [79]. The self-made stamps (S4 suhy dental a-silicone; Bisico, Bielefeld, Germany) were clamped into the bottom of the flexible membrane plates for 24 h.

### 4.4. RNA Expression Analysis

For analyzing gene expression, cells were isolated with TRIzol reagent (Thermo Fisher Scientific, Carlsbad, CA, USA). RNA isolation, cDNA synthesis, and quantitative PCR were performed as previously described [33,34,80]. Briefly, 1-bromo-3-chloro-propane (Sigma-Aldrich, St. Louis, MO, USA) and centrifugation were used for the separation of RNA. Cleaning of RNA was performed with an RNA Clean & Concentrator-5 kit (Zymo research, Freiburg, Germany). The quality and quantity of the RNA were measured with Nanodrop OneC (Thermo Fisher Scientific, Carlsbad, CA, USA). SuperScript IV Reverse Transcriptase (Thermo Fisher Scientific, Carlsbad, CA, USA) and Oligo(dt)18 primers (Thermo Fisher Scientific, Carlsbad, CA, USA) were used for cDNA synthesis. Quantitative PCR was performed with Luminaris Color HiGreen qPCR Master Mix (Thermo Fisher Scientific, Carlsbad, CA, USA) according to the manufacturer’s protocol and analyzed with qTOWER3 (Analytik Jena, Jena, Germany). Primer design was performed as previously described [33,34,80]. The quality and specificity of the primers were analyzed using melting curves and agarose gel electrophoresis. Dilution series of cDNA were used to calculate primer efficiency. Table 1 contains all information on the used primers. RPL22 and TBP were used as reference genes for data analysis according to the ∆∆CT method.

### 4.5. Co-Immunoprecipitation

For analyzing the interaction between GDF15 and ALK receptors, co-immunoprecipitation was performed in hPdLFs cultured to 75% confluency in 6-well plates, which were stimulated with 5 ng/mL rhGDF15 for 24 h. Cells were harvested in ice-cold phosphate-buffered saline (PBS) and with centrifugation. Co-immunoprecipitation was performed as previously described [81]. The following primary antibodies from Santa Cruz Biotechnology (Dallas, TX, USA) were used for precipitation: rat anti-ALK1 (sc-101556), mouse-anti-ACTR-I (ALK2; sc-374523), and rat anti-TGFβ-RI (ALK5; sc-101574). A GDF15-specific antibody (Abcam, Cambridge, UK) was used together with whole cell lysates as positive controls. Prior to protein detection using a semi-dry Western blot, the samples were stored at −20 °C.

### 4.6. Protein Preparation and Expression Analysis

For analyzing protein levels, either the samples from the co-immunoprecipitation or samples with ice-cold phosphate-buffered saline (PBS) and centrifugation-isolated hPdLFs were used. Further protein isolation and expression analysis by semi-dry Western blot was performed as previously described [81]. The following primary antibodies from Santa Cruz Biotechnology (Dallas, TX, USA) were used: rat anti-ALK1 (sc-101556, 1:100), mouse anti-ACTR-I (ALK2; sc-374523, 1:100), and rat anti-TGFβ-RI (ALK5; sc-101574, 1:100). The following secondary antibodies coupled to horseradish peroxidase (HRP) from Thermo Fisher Scientific (Carlsbad, CA, USA) were used: goat anti-mouse IgG HRP (#31430, 1:5000) and goat anti-rat IgG HRP (#31470, 1:5000).

### 4.7. Immunofluorescent Staining

To analyze cell characteristics, hPdLFs cultured on coverslips were fixed after specific treatment with 4% PFA for 10 min and washed three times with PBS containing 0.1% Triton™ X-100 (Merck Millipore, Burlington, MA, USA). Primary antibody incubation was performed for 3 h in a blocking solution consisting of PBS, 0.1% Triton™ X-100, and 4% bovine serum albumin, followed by three washing steps and subsequent secondary antibody incubation for 45 min in a blocking solution. DAPI (Thermo Fisher Scientific, Carlsbad, CA, USA; 1:10,000 in PBS) was applied for 10 min to stain cell nuclei. The following antibodies were used: rabbit anti-human p21 (18769S; Cell Signaling Technologies, Danvers, MA, USA; 1:100), rabbit anti-human Ki67 (ab15580; Abcam, Cambridge, UK; 1:500), and goat anti-rabbit-Cy5.

### 4.8. MTT Assay

The cell vitality was assessed using an MTT (3-(4,5-dimethylthiazol-2-yl)-2,5-diphenyl tetrazolium bromide) colorimetric assay (Sigma Aldrich, Taufkirchen, Germany) according to the manufacturer’s instructions and measured using the INFINITE M NANO microplate reader (Tecan Austria GmbH, Gröding, Austria).

### 4.9. Cell Death Assays

Trypan blue staining was performed on unfixed cells immediately after stimulation with rhGDF15. Cells were briefly washed with pre-warmed PBS, and 0.4% trypan blue solution was added to the PBS at a ratio of 1:2 for 5 min. After rinsing twice with PBS, microscopic images were captured immediately.

An ApopTag^®^ Fluorescein In Situ Apoptosis Detection Kit (Sigma Aldrich, St. Louis, MO, USA) was used according to the manufacturer’s protocol to detect apoptotic cells grown to 75% confluency on coverslips.

### 4.10. Senescence Assay (β-Galactosidase Staining)

To detect cellular senescence, 75% confluent hPdLFs cultured on coverslips were analyzed with the CellEvent™ Senescence Green Detection Kit (Thermo Fisher Scientific, Carlsbad, CA, USA) according to the manufacturer’s protocol, identifying active β-Gal using a fluorescein-based probe.

### 4.11. Alkaline Phosphatase Activity Assay

To analyze the activity of alkaline phosphatase, cultured hPdLFs were fixed with 4% PFA for 10 min, washed with PBS, and incubated with 1-Step™ NBT/BCIP Substrate Solution (Thermo Fisher Scientific, Carlsbad, CA, USA) for 90 min. After washing with PBS, the cells were directly imaged.

### 4.12. Alizarin Red Staining

Staining of calcium deposits to detect osteogenic differentiation was performed as previously described [82]. Briefly, cultured hPdLFs were fixed with 10% PFA for 10 min prior to staining with 40 mM Alizarin Red S (Merck Millipore, Burlington, MA, USA) for 20 min. Following a rinse with water, 10% acetic acid was used to dissolve the stained cells. Subsequent to heating at 85 °C for 10 min, cells were centrifuged at 20,000× *g* for 15 min, and the pH of the supernatant was neutralized with 10% ammonium hydroxide. OD405 was measured as duplicates using the Infinite M nano plate reader (Tecan Life Science, Männedorf, Switzerland).

### 4.13. THP1 Activation Assay

For visualization of the inflammatory response of stressed hPdLF, a THP1 activation assay was performed immediately after the application of mechanical forces as previously described [78]. To this end, 50 × 10^3^ non-adherent THP1 monocytic cells were stained with Celltracker CMFDA (Thermo Fisher Scientific, Carlsbad, CA, USA) and added to each well of a 24-well plate with cultured hPdLF. Non-adherent cells were removed using washing with prewarmed PBS, and coverslips were fixed with 4% paraformaldehyde (PFA) for 10 min. After washing with PBS, cell nuclei were stained with DAPI (1:10,000 in PBS, Thermo Fisher Scientific, Carlsbad, CA, USA) and coverslips were mounted for microscopy on objects slides.

### 4.14. Osteoclast Activation Assay and TRAP Staining

For the analysis of osteoclast differentiation induced by stressed hPdLFs, two-day phorbol 12-myristate 13-acetate (PMA, 100 ng/mL)-pre-stimulated macrophage-like THP1 cells were cultured in 96-well plates with the collected media supernatant of hPdLFs stressed with compressive force. For this purpose, supernatants of compressed hPdLFs were aliquoted immediately after the application of a 24 h compressive force, stored at −80 °C until use, and freshly thawed on each day of THP1 stimulation. Supernatants were applied 1:1 with fresh THP1 culture medium and changed daily. Subsequently, these cells were prefixed in 4% PFA for 10 min and then fixed for 1 min in 50:50 acetone/ethanol, air-dried, and stained for tartrate-resistant acid phosphatase (TRAP). A staining solution consisting of 0.1 mg/mL Naphtol AS-MX phosphate, 0.5 mg/mL Fast Red Violet LB salt, 1% N,N-dimethyl formamide in 50 mM sodium acetate trihydrate, 50 mM tartrate dehydrate, and 0.1% acetic acid (all Merck Millipore, Burlington, MA, USA) was applied for 60 min at 37 °C. Finally, cells were washed in PBS and directly imaged. Stimulation was performed with three independent collections of medium supernatant and analyzed on two coverslips per condition in each independent experiment.

### 4.15. Microscopy, Image Analysis, and Statistics

Immunofluorescent staining and the TUNEL and senescence assays, as well as the THP1 activation assay, were imaged with an inverted confocal laser scanning microscope TCS SP5 (Leica, Wetzlar, Germany). Fiji software I49.u (https://imagej.net/Fiji, accessed on 1 April 2017) was used for the image analysis. The fluorescence intensity of p21 was assessed as previously reported [83]. Briefly, in 270 cells per condition, mean grey values (MGVs) of the p21 staining were measured, and the background was subtracted for each measurement. MGVs were normalized to control conditions and presented as percent changes. For visual presentation, MGV was displayed as intensity as thermal LUT. Each experimental condition was analyzed at least in biological triplicates with two technical replicates per condition in each independent experiment. 

For statistical analysis and figure illustration, Graph Pad Prism 9 (https://www.graphpad.com, accessed on 1 February 2021) and Adobe Photoshop CS5 (https://adobe.com, accessed on 1 February 2013) were used. One-way ANOVA and post hoc test (Tukey) were used as statistical tests. The following significance levels were used: *p*-value < 0.05 */#/§; *p*-value < 0.01 **/##/§§; and *p*-value < 0.001 ***/###/§§§.

## 5. Conclusions

Orthodontic tooth movement is promoted with applied mechanical stimuli and relies on the crucial functions of PdL fibroblasts regarding the modulation of tissue and bone remodeling. Here, we could demonstrate that GDF15 alters the cell fate of PdLFs in an anti-proliferative and pro-osteogenic manner and further affects the mechanoresponse of PdLFs. These findings provide a link for future clinically focused research to evaluate how orthodontic treatments are affected by elevated GDF15 blood levels, as seen in patients with advanced age and pathologic conditions such as inflammation, myocardial ischemia, and cancer [43].

## Figures and Tables

**Table 1:** qPCR primer sequences of human genes indicated in the 5′-3′ direction. bp, base pairs. Length, amplicon length.

Gene	Gene Symbol	NCBI Gene ID	Primer Sequence	Length
Activin A receptor like type 1	*ACVRL1* *(alias ALK1)*	94	fw: GTGGAGTGTGTGGGAAAAGG rev: CATGTCTGAGGCGATGAAGC	180 bp
Activin A receptor type 1	*ACVR1* *(alias ALK2)*	90	fw: GCATTCCCAGAGCACCAATC rev: GGCCACTTCCCACAAAACAA	166 bp
Alkaline Phosphatase	*ALPL*	249	fw: ACTGCAGACATTCTCAAA rev: GAGTGAGTGAGTGAGCA	190 bp
Interleukin 6	*IL6*	3569	fw: CATCCTCGACGGCATCTCAG rev: TCACCAGGCAAGTCTCCTCA	164 bp
Interleukin 10	*IL10*	3586	fw: AGCCATGAGTGAGTTTGACA rev: AGAGCCCCAGATCCGATTTT	141 bp
Interleukin 1 receptor antagonist	*IL1RN*	3557	fw: GATGTGCCTGTCCTGTGTCA rev: ACTCAAAACTGGTGGTGGGG	146 bp
Prostaglandin-endoperoxide synthase 2	*PTGS2* *(alias COX2)*	4743	fw: GATGATTGCCCGACTCCCTT rev: GGCCCTCGCTTATGATCTGT	185 bp
Ribosomal protein L22	*RPL22*	6146	fw: TGATTGCACCCACCCTGTAG rev: GGTTCCCAGCTTTTCCGT TC	98 bp
RUNX family transcription factor 2	*RUNX2*	6146	fw: CCCACGAATGCACTATCC rev: GGACATACCGAGGGACA	120 bp
TATA-box binding protein	*TBP*	6908	fw: CGGCTGTTTAACTTCGCTTCC rev: TGGGTTATCTTCACACGCCAAG	86 bp
TNF receptor superfamily member 11b	*TNFRSF11B* *(alias OPG)*	4982	fw: GAAGGGCGCTACCTTGA	142 bp
			rev: GCAAACTGTATTTCGCTC	
TNF Superfamily Member 11	*TNFSF11* *(alias RANKL)*	8600	fw: ATCACAGCACATCAGAGCAGA rev: TCACTTTATGGGAACCAGATGGG	160 bp
Transforming growth factor beta receptor 1	*TGFBR1* *(alias ALK5)*	7046	fw: AAAACTTGCTCTGTCCACGG rev: TGCCAGTCCTAAGTCTGCAA	157 bp

## Data Availability

The datasets used in this study are available upon reasonable request from the corresponding author.
